# A conceptual model for studying the immersive mobile augmented reality application-enhanced experience

**DOI:** 10.1016/j.heliyon.2022.e10141

**Published:** 2022-08-12

**Authors:** Vo Kim Nhan, Le Thanh Tam, Ho Tien Dung, Nguyen Thanh Vu

**Affiliations:** a Candidate of University of Economics Ho Chi Minh City, Vietnam; b Lecturer of Tien Giang University, Vietnam; c The Department of Commercial Banking, School of Banking and Finance, National Economics University, Vietnam; d University of Economics Ho Chi Minh City, Vietnam; e Nguyen Tat Thanh University, Vietnam

**Keywords:** Mobile applications, MAR apps, immersive experience, virtual technologies

## Abstract

**Purpose:**

This study aims to develop and propose an integrated conceptual framework that illustrates how emerging technologies such as mobile augmented reality applications (MAR apps) stimulate a user's immersive MAR app-enhanced experience—a human psychological state of being engaged and engrossed in a virtual environment—which in turn facilitates user responses.

**Design/methodology/approach:**

This study draws on a literature review of related fields to develop a theoretical model showing the centrality of the immersive MAR app-enhanced experience.

**Findings:**

A conceptual model that explicates the selected antecedents and outcomes of the AR-enhanced immersive experience is proposed. The findings suggest that the traits of both the user (mental imagery, personal innovativeness) and the device (simulated physical control, environmental embedding) facilitate the immersive MAR app-enhanced experience. Moreover, the immersive MAR app-enhanced experience is identified as a key driver of customer emotions, values and behavioral responses.

**Originality/value:**

The integrated conceptual model provides scholars and practitioners with a general picture of the main factors affecting the immersive AR-enhanced experience, as well as the benefits available to firms; thus, theoretical and practical implications are delineated.

**Paper type:**

Conceptual paper.

## 1 Introduction

Immersive technologies such as augmented reality (AR) have been applied to diverse areas of marketing (Wedel et al., 2020). In the tourism context, AR technologies have been deployed in various activities across the customer journey which offer potential tourists a compelling image of particular tourism destinations in the pre-visit stage (Bogicevic et al., 2021; Hudson et al., 2019), or they can enhance the theme park experience for users during their visit (Wei, 2019) by giving them a sense of “being there” as well as having a “real” and “authentic” experience. In the general business and marketing fields, applying technological innovations to enhance customers' feeling of immersive experience is the primary means for helping firms to achieve higher performance through influencing the positive emotions and values of consumers, eventually changing their behavioral responses (Heller et al., 2019a). In spite of the significance of AR, a comprehensive literature review reveals relatively little scholarly marketing attention to the nature of mobile and emerging technologies or to feelings of immersion in virtual environments (Hilken et al., 2018). In the retail setting, there remains a lack of studies investigating the dimensions of the immersive MAR app-enhanced experience. To the best of our knowledge, there is no comprehensive review of the existing literature to integrate the antecedents and outcomes of the immersive MAR app-enhanced experience. There is thus a call for a conceptual model of the immersive MAR app-enhanced experience that promises to provide important theoretical and managerial implications pertaining to the applications of advanced technologies such as MAR apps.

## 2 Conceptual background of emerging technological advancement

### 2.1 Augmented reality

AR is a type of technology that enables adding an extra layer of virtual information in the real world (Loijens et al., 2017). It allows users to experience the real world with virtual objects superimposed on them through a computer or smartphone camera. In light of the growing applications of AR in marketing, marketers focus on creating, communicating, and distributing digital affordances in the physical environment with the aim of enhancing customers' immersive experiences to guide their decision-making (Chylinski et al., 2020; Hilken et al., 2018). However, many firms lack the knowledge and capabilities to improve interactive customer experiences that could reach and comprehensively engage with their customers (Chylinski et al., 2020). This indicates a clear need for a more comprehensive understanding of how AR marketing creates and delivers customers' immersive experiences in a way that is different from other marketing approaches. The comprehensive framework presented in this paper will provide deeper insight into customer experiences, and attitudinal and behavioral responses in AR environments.

### 2.2 Mobile applications (mobile apps)

Mobile apps have emerged as one of the most powerful, ubiquitous and convenient products and service delivery channels of contemporary life (Grewal et al., 2017; Mclean et al., 2018). Shankar and Balasubramanian (2009) contended that mobile app marketing is “the two-way or multi-way communication and promotion of an offer between a firm and its customers using a mobile medium, device or technology”. Mobile apps are a tool for marketers, sellers and retailers to advertise and sell their products and services. Customers can receive more information about products by using mobile apps in virtual environments with advanced technologies, such as virtual reality or augmented reality. Shankar et al. (2010) advocated that mobile apps have an impact on customer attitude and behavioral responses. Marketers allocate their budgets to marketing activities that enhance customer experience through mobile apps (Mclean et al., 2018). Mobile apps are considered a popular and convenient technological tool because of their portable functions (Hur et al., 2017). Mobile apps not only bring certain technological benefits to the customer experience but also reduce uncertainty in purchasing decisions, which in turn leads to an increase in the company's brand image (Hur et al., 2017). Marketers and retailers have utilized mobile apps to enhance customer value. Moreover, immersive experiences using MAR apps in retailing settings and their effects on user responses are relatively less understood. There is, therefore, a call for investigating the immersive experience, in particular its driving forces and how exactly it influences customers' emotions, values, and responses.

### 2.3 Mobile augmented reality applications (MAR apps)

Mobile apps are one of the impressive tools in mobile commerce, used by more than 49% of businesses in Vietnam (Tuu et al., 2021). According to Chen et al. (2021), MAR apps have become a crucial technology for “virtual try-on” in retail settings during the COVID-19 pandemic, such as clothing, footwear, cosmetics, and even furniture in customers' homes (e.g., IKEA, Dulux). There are several benefits to using MAR apps, such as reducing uncertainty in decision making, increasing shopping intention, enhancing customer loyalty, and building relationship between customers and firms (Chen et al., 2021). Companies can develop MAR apps with additional information for their customer. Recent studies have provided some early evidence that MAR apps can support customers by providing more information about virtual products (Dacko, 2017; Hilken et al., 2020); as such, companies have developed MAR apps with additional information about AR products in order to provide more benefit for their customers (Mekni and Lemieux, 2014). Moreover, Huang and Liao (2015) stated that augmented reality technology provides customers with richer experiences and greater enjoyment. Hence, experiencing try-on virtually in MAR apps is so enjoyable that customers are willing to experience this, even at a great cost (Shin, 2019). Given the brief review above, it would be valuable to study more conclusively whether users accept using MAR apps to attain added value before making purchase decisions.

## 3 Literature review on immersive experiences

### 3.1 A review of prior immersive experience studies

Researchers have studied customer immersion or the immersive experience in different settings such as tourism (Tsai, 2019; Hudson et al., 2019), education (Radianti et al., 2020), and retailing (Peukert et al., 2019; Song et al., 2019), with its potential dominance for both customers and business firms. From a technological perspective, immersion is utilized to show a level of a device's functionality (Flavián et al., 2019a) in which augmented reality and virtual reality are immersive and emerging technologies (Suh and Prophet, 2018). From a psychological perspective, immersion refers to a level of user experience and is a multi-dimensional construct consisting of engagement, engrossment, and total immersion (Brown and Cairns, 2004; Carù and Cova, 2006). Later on, Hilken et al. (2018) contended that the immersive user experience of using augmented reality technology is affected by their personality. In addition, Witmer and Singer (1998) imparted that immersion refers to a human psychological state of “being enveloped by, included in, and interacting with an environment that provides a continuous stream of stimuli and experiences.” From a social perspective, customer immersion is understood as a user experience process of “being plunged in a thematised and secure spatial enclave where they can let themselves go” (Carù and Cova, 2006). From an experience economy perspective, Pine et al. (1999) argued that immersion is “becoming physically (or virtually) a part of the experience itself.” Moreover, Agarwal and Karahanna (2000) articulated that user immersion is a dimension of cognitive absorption that can enhance user attitudes and lead to behavioral responses. In recent studies, there have been two ways of classifying the dimensions of customer immersion: engrossment, engagement and total immersion (Georgiou and Kyza, 2017) or cognitive, affective and social levels (Ali, 2022). *Engagement* refers to users' psychological state in which they can “access the activity”, and then “invest time and effort to attend to the activity”. *Engrossment* refers to users' psychological state in which they may be able to “become further involved with the activity”. *Total immersion* means users' psychological state in which they are totally absorbed and immersed in their surroundings. *Utilitarian* is considered to be customers' perceptions toward enabling the efficient and practical use of MAR apps for their experience. These perceptions provide the extent to which MAR apps can create immersive experiences with realistic, helpful, creative, efficient, innovative, practical hands-on features. *Hedonic* refers to effective constructs such as the playful emotions and pleasurable feelings of the immersive MAR app-enhanced experience. *Social* refers to customers' perceptions toward enabling the use of MAR apps for their experience interactively and collectively.

Common to all these definitions is the idea that the immersive MAR app-enhanced experience is understood to be a customer's immersive experience related to MAR apps consisting of three dimensions of engagement, engrossment and total immersion from a psychological perspective (Georgiou and Kyza, 2017). In Table 1, we review some current studies related to user experience and reveal some research gaps.

**Table 1:** Summarize customer experience using AR apps in marketing settings.

Studies	AR types	Conceptualization of customer experience	Antecedents	Outcomes	Findings
Huang and Liao (2015)	online fitting through ARIT (augmented-reality interactive technology) on websites	Presence	–	Sustainable Relationship Behavior	Using augmented-reality interactive technology (ARIT), presence have impact on sustainable relationship behavior through mediating variables (e.g., values) and moderating variable (consumers' innovativeness)
Javornik (2016)	IKEA Place app & website	Flow	AR characteristics (Augmentation, control, responsiveness)	Affective responses (application attitude, brand attitude), Conative responses (Thoughts), Behavioral intentions (Purchase intentions, revisit intentions, recommendation intention)	Examine the differences in consumer responses to media characteristics of AR apps and non-AR apps
Kim and Hyun (2016)	OVJET AR app	Telepresence & Usefulness	System quality, information quality, service quality	AR reuse intention	Two models to determine whether telepresence or usefulness can mediate the relationship between three types of AR quality and the intention to reuse AR
Huang and Liao (2017)	ARIT in an online clothes fitting	Flow experience (concentration, playfulness, time distoration, exploratory behavior)	Multisensory features (self-location, haptic imagery) and Decorating psychological states (sense of body ownership, ownership control, self-explorative engagement)	Satisfaction, spend more time on ARIT	Haptic imagery and sense of self-location positively influenced perceived sense of body ownership, perceived ownership control, and self-explorative engagement. Flow experience has a positive impact on satisfaction and spend more time on ARIT
Yim et al. (2017)	AR-based vs. web-based Ray-ban and TISSOT	Immersion	Interactivity, Vividness, Media novelty	Purchase intention	AR generates greater novelty, immersion, enjoyment, and usefulness, resulting in positive attitudes toward medium and purchase intention Immersion plays mediating role of the relationship between interactivity/vividness and usefulness and enjoyment
Poushneh and Vasquez-Parraga (2017)	Ray-Ban app/website	Retail user experience	Augmented reality	User willingness to buy, user satisfaction	AR shapes UX, and that UX influences user satisfaction and user's willingness to buy; UX is formed as a third-order formative construct from four user experience characteristics: pragmatic quality, aesthetic quality, hedonic quality by stimulation and hedonic quality by identification
Hilken et al. (2017)	L'Oreal's AR virtual mirror (web, app); Mister Spex (web/app)	Spacial presence	Simulated physical control (SPC), Environmental embedding (EE)	Behavioral intensions (purchase, WOM)	A conceptual framework showing AR-based spacial presence affected by simulated physical control and environmental embedding, enhancing behavioral intentions, mediated by hedonic, Utilitarian value, decision comfort, moderated by SOP and APP
Hilken et al. (2018)	–	Realism of the experience (cognitive and emotional fit, fidelity, immersion, spacial presence)	AR variables (Embedding, Embodiment, Extention)	Decision making, behavioral intentions, brand and application perception	A conceptual framework in which 3 AR variables (embedding, embodiment and extention) lead customer experience, evaluation of experience and their consequences, analysing moderating role of contingency factors
Bonetti et al. (2018)	–	Customer experience (spacial presence, flow, immersion, mental imaging)	Media characteristics and Media quality	Decision making behavioural intentions and Attitudinal Outcomes	A framework on customer behavior towards AR in online retailing
Yim and Park (2019)	Ray Ban app/website	product involvement	Media features (perceived interactivity, media irritation, medianovelty)	Adoption intention	Analyzing the difference between AR &VR Moderating role of body image in consumer responses between AR with traditional website
Perannagari and Chakrabarti (2019)		Cognitive (flow &value) & affective (attitude)responses	Design features (augmentation quality & media characteristics)	Behavioral response (BI)	A summary of literature by thematic analysis Designing a conceptual framework to explain the decision-making process of retail customers.
McLean and Wilson (2019)	Amazon, ASOS & IKEA apps	Brand engagement (cognition, affection&activation)	AR attributes (Interactivity, Vividness, Novelty)	Satisfaction with customer experience & Brand use intention	Revealing a new set of AR attributes & technology attributes Brand engagement has a positive impact on satisfaction and use intention, analysis moderating role of purpose of use in the model
Song et al. (2019)	Formex try-on watches	Immersion	Environmental embedding (EE) and Stimulated physical control (SPC)	Decision comfort	Immersion was affected by Environmental embedding and Stimulated physical control; explaining mechanism how AR experiences (EE &SPC) induce feeling of ownership, lead to decision comfort, the moderating role of prior AR try-on experience evoke weaker immersion than without prior experience.
Jessen et al. (2020)	IKEA Place (website/app)	Creative customer engagement (customer engagement, customer creativity)	Use of AR	Anticipated satisfaction	AR enables creative customer engagement, in turn, offers intrinsic satisfaction and moderating of assessment orientation.
David et al. (2021)	Amazon shopping app	Customer experience (satisfaction)	Service quality & visual quality (aesthetics & position relevance)	Recommendation intention	Explaining the influence of visual and service quality on user satisfaction toward the app and the impact of user satisfaction on recommendation intention
Heller et al. (2021)	–	Spacial presence	Visual appeal, information fit-to-task	Service reuse likelihood, WOM	Proposing a technology-enabled engagement process (TEEP) of AR service
Nikhashemi et al. (2021)	Atleast 2 AR apps (Gap, IKEA, Amazon)	Brand engagement&, psychological inspiration	AR quality, Interactivity, Vividness, Novelty	Continuous intention to use AR app, willingness to pay price premium (WPPP)	A symmetric approach of the chain from AR attributes on CI&WPPP through U&H benefits and moderating role of customisation
Wang et al. (2021)	YouCam Makup	Spacial presence	Consumer perception of MAR service (interactivity, vividness, augmentation, aesthetics)	PI	Virtual contents (interactivity, vividness, augmentation, aesthetic) affect purchase intention mediated by spacial presence, flow experience, and decision comfort Moderating role of Individualism &fashion innovativeness
Qin et al. (2021)	IKEA Place app&Ray-Ban Virtual Try-on app	Cognition (Virtual presence, experimental value, shopping benefits, perceived value)	–	Conation (continue use intention, PI)	Consumer's cognitive evaluation using MAR apps stimulates their affective responses, which create conative responses.
Kowalczuk et al. (2021)	IKEA Place AR app or the IKEA mobile website	Affective responses (Immersion, enjoyment, product liking)	AR characteristics (Interactivity, system quality, product informativeness, congruence)	Behavioral responses (reuse intention, PI)	Behavioral responses (purchase intention) are formed by affective (enjoyment, immersion, product liking) and cognitive (choice confidence, media usefulness) responses to the AR characteristics (system quality, interactivity, reality congruence, product informativeness)
Plotkina et al. (2021)	Converse, Zara, Joy Walks	Perceived AR app experience (pleasure, Playfulness)	AR type (goal, location)	Perceived brand personality (excitement, sincerity, competence and sophistication)	AR types receive more positive evaluations and lead customers to perceive brand personality, in which playfulness determine consumer attitude toward AR app
Barhorst et al. (2021)	Crimes wine brand	Flow	Interactivity, vividness, novelty	Satisfaction AR experience	Determining investment in AR technologies is warranted by exploring flow in both an AR and a traditional shopping context Examining flow affecting positively consumer outcomes
Yuan et al. (2021)	Zara' VTO	Flow experience	Perceived informativeness, perceived aesthetics, perceived novelty, parasocial relationship	Psychological ownership	Perceived informativeness, perceived aesthetics, perceived novelty, and parasocial relationship all positively influence flow experience, in turn, to feel ownership, moderated by brand attachment
Huang and Liu (2021)	Kinect	Humanizing digital experiences (Anthropomorphism, intimacy, self-representation)	a 360° AR panorama	Green destination brand love (place identity, affective attachment, compatibility)	Examining the antecedents and consequences of humanizing the digital experience in a virtual tourism context
Huang (2021)		Restorative experience (coherence4, compatibility5, being away5, fascination8)	Environment embeding3 & situated physical control3	Actual willingness to pay a price premium	
J. V. Chen et al. (2021)	AR mobile shopping apps (flowers)	Local presence	Vividness& Spacial accuracy	Urge to buy impulsively	Examining media characteristics (vividness and spatial accuracy) Affecting the feeling of local presence - examine consumer perceptions (arousal and perceived diagnosticity) affecting consumer's urge to buy impulsively
Arghashi and Yuksel (2022)	“Wanna Kicks” AR app	Flow	AR attributes (interactivity, inspiration)	Brand usage, brand attitude	Flow experience stimulated by AR attributes can generate favourable attitudes and trust, engagement, in turn, to crate brand usage and brand attitude and moderated by perceived usefulness
Chen et al. (2022)	Wanna Kicks and FitGlasses apps	Customer experience	–	Continuance intention, purchase intention, customer engagement	A model to evaluate the antecedents and consequences of AR marketing activities by adding customer experience, continuance intention, purchase intention and customer engagement
This study	MAR apps	Immersive experience	User traits & device traits	Positive emotions, Customer values, behavioral responses	Proposing a conceptual model of MAR apps-enhanced immersive experience Providing theoretical and managerial implications into the applications advanced technologies

Notes: Augmented Reality (AR), Perceived usefulness (PU), Perceived ease of use (PEOU), Perceived security (PS); Perceived enjoyment/entertainment (PE), Perceived risk (PR), Attitude towards using (ATU); purchase intention (PI); Virtual Try-on (VTO); Utilitarian Value (U); Hedonic value (H); Stimuli-Organism-Responses (S–O-R); The technology acceptance model (TAM), The Theory of Reasoned Action (TRA); augmented-reality interactive technology (ARIT).

Source: author's summarization.

## 4 Methodology

### 4.1 Criteria for choosing previous studies in the literature review

There are some criteria used in this study for choosing suitable publications, listed as follows. *Centrality of topic*: the customer experience should be the focus of the article, meaning that we excluded articles that are not relevant to customer experience. *MAR apps context:* The subjects of the study needed to engage with virtual technologies like augmented reality. This means we excluded studies without virtual technologies. *Year of publication*: We included all studies that were published in the period from 2010 to 2020, as the most recent literature review on the immersive experience only includes studies published in recent years. *Language*: We only included studies that were written in English, meaning that published works written in other languages were excluded from this study.

### 4.2 Literature search

**Figure 1:**
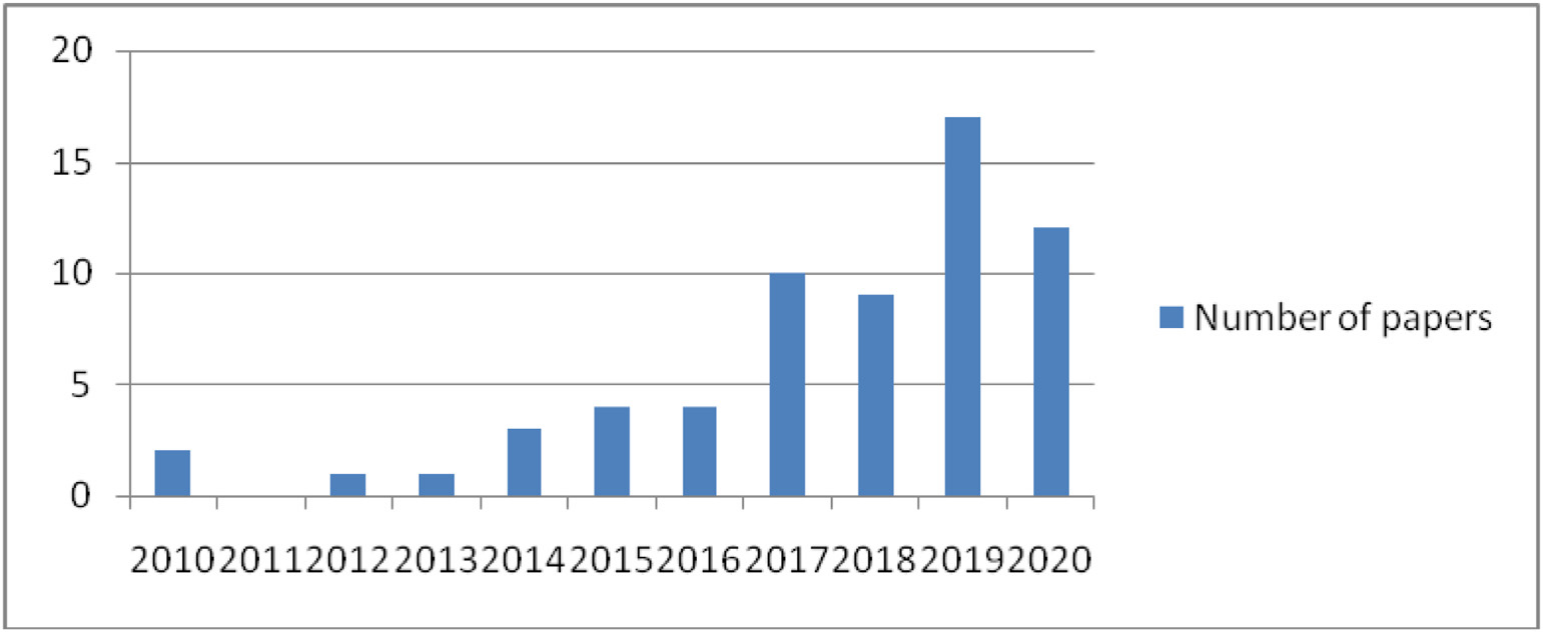
Papers related immersive experience on Web of science 2010–2020. Source: Web of Science.

**Figure 2:**
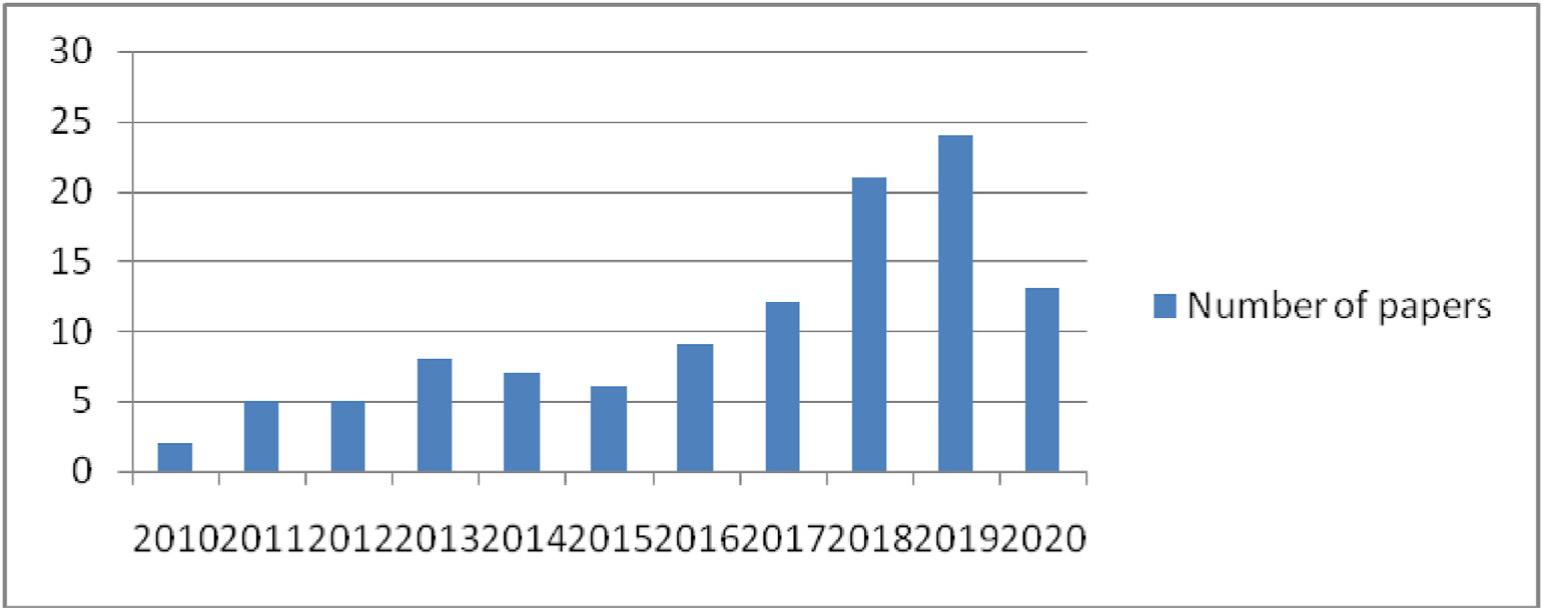
Papers related immersion on Scopus web in the period 2010–2020. Source: Website of Scopus, 2020.

In order to find relevant studies that could then be comprehensively integrated into a conceptual model of the immersive MAR app-enhanced experience, the authors carried out a procedure comprised of four stages. Firstly, we searched on the Web of Science and Scopus websites using the keyword “immersi∗” combined with (“virtual reality” or “augmented reality” or “mobile augmented reality applications” or “virtual technolog∗”). We only focused on recent studies from 2010 to 2020. The works included in this review were papers from international or national conference proceedings, journals, book chapter reviews, etc. The initial search resulted in 207 publications. Secondly, after removing all duplicates and excluding papers from international or national conference proceedings, book chapter reviews, etc., we kept peer-reviewed journal articles only. Later on, by carefully reading the titles, abstracts, keywords and full texts, we manually chose 59 theoretical and empirical articles on the topic of the immersive experience and its relation to augmented reality technology (see Figure 1, Figure 2). Thirdly, we summarized articles that cited the immersive experience, including studies cited by year, antecedents and outcomes of immersive experience, theories, AR types, etc. Fourth, we reached out to six experts in the fields of technology, tourism and marketing to ask them to check our list of suitable publications in order to update new publications and to point out studies that we might have missed. For instance, some new keywords were added such as “virtual try-on” and “virtual technologies”. Finally, 30 high-ranking articles were chose as illustrated in Table 1.

### 4.3 Selection process for including articles

The initial search resulted in 207 publications on the Web of Science and Scopus websites, yet ultimately only 30 peer-reviewed articles met our criteria, as mentioned earlier. After that, all the authors carefully read every single article to sort out antecedents, outcomes, mediators, and moderators of the relevant research models. Based on such a comprehensive review, a conceptual model of the immersive MAR app-enhanced experience is proposed and discussed in the following section.

## 5 Proposing conceptual model

### 5.1 Antecedents of the immersive experience

We reviewed current studies related to users' experiences to help reveal the key antecedents and outcomes of the immersive experience (see Table 1). In exploring digital media engagement, the existing literature has employed self-traits such as mental imagery and personality as antecedents of the immersive experience in the online shopping and tourism domains (Bogicevic et al., 2019). In addition, device traits can be categorized into environmentally embedding and simulating physical control (Hilken et al., 2017). In the current conceptual framework, we propose that these self-traits (mental imagery and personal innovativeness) and device traits (environmental embedding and simulated physical control) enable the feeling of immersion within a virtual experience.

#### 5.1.1 Mental imagery

Mental imagery is defined as “a process by which visual information is represented in the working memory” (Heller et al., 2019a). AR apps can provide a clear, detailed representation of an image (namely, imagery vividness) through the combination of the real world and virtual world (McLean and Wilson, 2019). Vividness is considered to be the quality of product presentations (Yim et al., 2017) or “the clarity with which the individual experiences an image and which reflects its quality” (Gavilan et al., 2014). It also enables consumers to mentally have a “try before you buy” experience, thereby resulting in an enhanced long memory of relevant product information. In online settings, augmented reality technology enables customers to enhance the vividness of their experience (González et al., 2021). In addition, imagery quality is the number of images in a customer's mind while processing information (Babin and Burns, 1998). After a try-on experience, customers can evoke many images in their minds about how the products display on mobile AR apps. Moreover, imagery elaboration is defined as “the activation of information in the production of mental images beyond what is provided by the stimulus” and it is created by establishing integrations between information provided and that stored in the long-term memory (Babin and Burns, 1998). It means new information is stored in individual memory and that information is provided and activated by the stimulus in information processing. After a virtual try-on experience via AR apps as a stimulus evoked by previous image-related experiences, users become more familiar with previous images in their thoughts and knowledge, which in turn invokes deeper elaboration. Thus, in this study, mental imagery consists of three components, including imagery vividness, imagery quantity and imagery elaboration (Babin and Burns, 1998; Gavilan et al., 2014).

Mental imagery plays an imperative role in the information processing of the human brain (Park and Yoo, 2020). It also enables customers to have the ability to imagine and activate stored information as mental images in which they can make pictures appear in their minds, evoke previous image-related experiences, and then have an impact on affective and cognitive responses (Park and Yoo, 2020). Bogicevic et al. (2019) supported that mental imagery has an impact on a sense of presence, which is the feeling of being there through virtual reality technology in a tourism setting. Thus, we expect that mental imagery as a personality trait has a positive impact on the immersive experience of using MAR apps as follows:

Proposition 1 Mental imagery has a positive effect on the immersive experience.

#### 5.1.2 Personal innovativeness

There are two types of innovativeness, including innate innovativeness and domain-specific innovativeness (Agarwal and Prasad, 1998). Innate innovativeness is also called a personality trait or personal innovativeness, reflecting a tendency to try out new information, stimuli or experiences. Domain-specific innovativeness is defined as an individual tendency to learn or adopt new products/services with a specific interest. As mentioned above, personal innovativeness is considered an individual trait reflecting their willingness to try out new things, especially new technology (Agarwal and Prasad, 1998). A person with high innovativeness has strong intentions to try out virtual technologies such as AR mobile apps. Individuals with high personal innovativeness are also believed to explore novelty and are motivated to try out new technology to gain more knowledge about products/services on MAR apps. Previous studies argued that innovators have the ability to imagine, understand and get benefits from new technologies (Krey et al., 2019). Customers with high innovativeness can look for new interactive technologies to achieve their experience; thus, they focus on new technologies that can help them accomplish their tasks. In contrast, customers with low cognitive innovativeness lack the ability to imagine and interact with new technologies such as AR apps, and thus they are insensitive to the features of new technologies. Therefore, personal innovativeness can lead customers to try out and experience MAR apps, in turn adopting continuing usage regarding that technology. Based on the discussion above, we expect personal innovativeness to have an impact on the immersive experience.

Proposition 2 Personal innovativeness has a positive effect on the immersive experience.

#### 5.1.3 Environmental embedding

Two dominant technological features of AR consist of simulated physical control and environmental embedding to meet customer demands (Hilken et al., 2017). The environmentally embedded concept is considered as the interaction of digital and virtual content (e.g. product information, product images) with the physical environment (e.g. the user's body as face, hands or living rooms) (Hilken et al., 2017). When we see our own images in a mirror, we think that this is our body as we are seeing, and when we move and control our body, our image in the mirror will respond accordingly. Before making a purchase, it is difficult for us to imagine how the company's products are suitable for us unless we physical try them on (Heller et al., 2019b). It is argued that customers are willing to pay more money for products having MAR app functions. Thus, MAR apps have been developed by firms to create try-on experiences for their customers before making purchasing decisions. For example, some customers can virtually try-on items to find the best look in the virtual clothing department using only their smartphone-installed MAR apps. Products which are embedded in a virtual environment can provide more information and enhance the user experience. Based on AR features, customers are ready to have a try-on and feel being there. Thus, the hypothesis is proposed as follows:

Proposition 3 Environmental embedding has an impact on the immersive experience.

#### 5.1.4 Simulated physical control

Simulated physical control can be considered a “real embodiment” (Hilken et al., 2017). In other words, a customer can simulate a product (e.g. move, rotate). Embodiment can be considered as the degree of integration of the technology with the human body; it shows how the customer's brain represents his/her body (Tussyadiah et al., 2018; Orús et al., 2021; Flavián et al., 2019a). Because of not touching products directly while shopping online, customers tend to experience bodily interactions (Gatter et al., 2022); they feel like they are controlling the virtual products, and they actually own the virtual products inside their bodies through a mobile camera. Thus, we anticipate the following hypothesis:

Proposition 4 Simulated physical control has a positive effect on the immersive experience.

### 5.2 Outcomes of immersive experience

Contextual stimuli initiate human feelings, perceptual and thinking activities such as consumer emotions (Chylinski et al., 2020), values (Hamilton et al., 2016), and behavioral responses (Kowalczuk et al., 2021).

#### 5.2.1 Consumer emotions

One's emotional state is defined as an “internal and subjective experience by an individual of a complex behaviour of physical and mental changes in reaction to some situation” (Li et al., 2022). Consumer emotions consist of positive and negative emotional states which are operationalized into the three dimensions of dominance, pleasure and arousal. Using mobile apps, consumers believe that they can get more useful information quickly and conveniently (Wolfinbarger and Gilly, 2001). As a result, consumers feel engaged with virtual products using mobile apps. Furthermore, immersive experiences can provide comfortable stimuli for customers and thus make them feel more positive emotions. Thus, we suggest the following hypothesis:

Proposition 5 The immersive experience has a positive effect on positive emotions

#### 5.2.2 Customer values

Hamilton et al. (2016) studied the relationships between the interaction/immersive experience and customer value in social media settings. In this study, customer value is related to customer lifetime value, influencer value, and knowledge value. The immersive interaction experience is a psychological state in which consumers are fully engrossed within the social media environment and exclusively fixated upon brand interaction (Novak et al., 2000). Customer value includes utilitarian value, hedonic value and social value. This study also identifies that the effects of the immersive experience on utilitarian value perceptions are greater for customers who use verbal rather than visual information processing, and the positive effect of customer value on decision comfort is attenuated by customers' privacy concerns. Nevertheless, we can propose the following hypothesis:

Proposition 6 The immersive experience has a positive effect on customer values

#### 5.2.3 Behavioral responses

Extending the previous discussion in Proposition 6, we expect the immersive MAR app-enhanced experience will lead to behavioral intentions. Prior studies' show that affective factors (i.e. liking, enjoyment) have a relationship with behavioral intention (Yim et al., 2017). According to Kowalczuk et al. (2021), affective factors (i.e. immersion, presence) also have an impact on behavioral responses (i.e. adoption intention) towards MAR apps. Before making shopping decisions, customers expect to virtually try on products based on MAR apps. Therefore, we propose the following hypothesis:

Proposition 7 The immersive experience will lead to behavioral intentions toward using MAR apps

### 5.3 A conceptual model of the immersive experience

**Figure 3:**
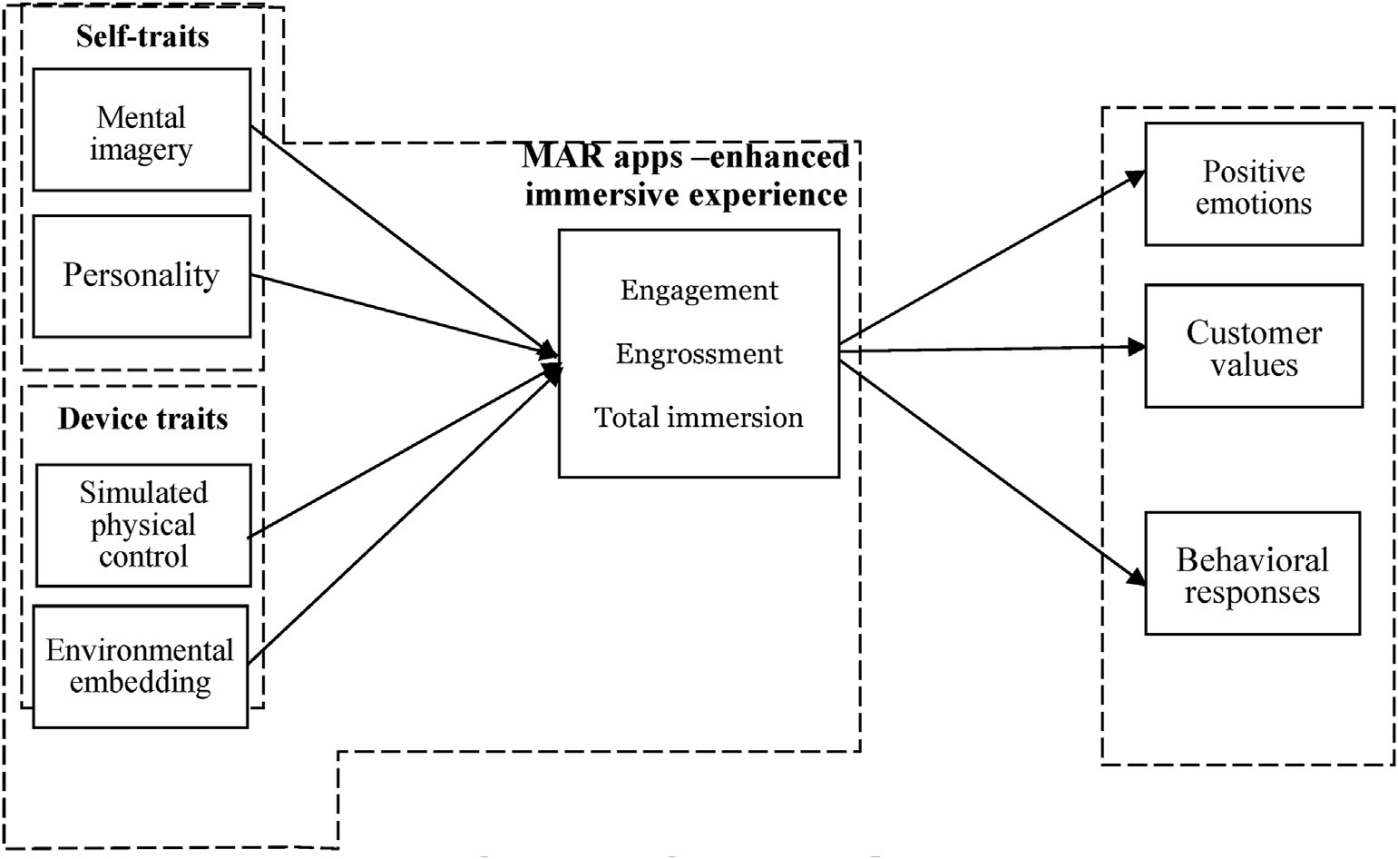
Integrated conceptual model.

Prior studies (Renner et al., 2019; Ahmad and Abdulkarim, 2019; Hilken et al., 2017) reviewed that not only individual traits (e.g. mental imagery, personal innovativeness) but also device traits consisting of environmental embedding and simulated physical control have relationships with the immersive customer experience. Recent researchers emphasize that virtual technologies (i.e., VR and AR) can provide more information and stimulate customer immersive experience (Kim et al., 2017) and they are also expected to affect customer emotions and decision-making evaluations (Dacko, 2017; Hilken et al., 2017). According to Javornik (2016), MAR apps can embed virtual content into real environments; thus, several companies have applied this emerging technology in creating virtual products. However, the immersive experience with MAR apps and its impact on customer responses are still neglected. The literature review reveals that immersive experiences can help achieve higher performance by influencing customers' positive emotions (e.g., pleasure, arousal, dominance), customer values (e.g., utilitarian value, hedonic value, social value) and eventually changing their behavioral responses (Novak et al., 2000; Yim et al., 2017). In summary, the current work seeks to theoretically incorporate self-traits and device traits as antecedents of an immersive experience, which in turn leads to the outcome variables of customer emotions, values and behaviors as described in Figure 3.

## 6 Discussions and implications

### 6.1 Discussions

Consistent with prior studies (Hilken et al., 2022), we find MAR apps an emerging technological advancement that determines the immersive customer experience and then their emotional and behavioral responses. This study has some significant contributions. Firstly, previous studies have utilized only one product or an AR app for their research in retail settings (e.g., Song et al., 2019; Jessen et al., 2020); this study reviews the applications of AR technology in marketing contexts from a number of countries and intends to propose a comprehensive conceptual model for AR apps. *Secondly*, previous studies have discussed customer experiences through AR features and the motivations of customers using AR apps (Yim et al., 2017; Poushneh, 2018; Hsu et al., 2021), but few studies have specifically examined the impact of customer traits as personal innovativeness and mental imagery on the immersive experience. *Thirdly*, this study has highlighted the importance of the immersive experience or psychological state engaged in technological devices from a psychological perspective (e.g. Song et al., 2019), while other studies have focused on the characteristics of virtual devices in social or technological perspectives. In previous studies (Flavián et al., 2019b; Hilken et al., 2018), the immersive experience is only applied in education and game contexts in developed countries; there have been few studies investigating the usage of AR apps for marketing purposes. *Last but not least*, by investigating the immersive MAR app-enhanced experience, scholars can understand why consumer experience plays a crucial role in the field of technology-based marketing. This proposition is in compliance with the view of Qin et al. (2021) that customers' affective responses would enrich their responses after using MAR apps.

In summary, the current work contributes to the existing literature with a comprehensive theoretical framework to enhance customer emotions, values and behaviors by incorporating the responses of customers into a model of immersive experience, self-traits and device traits in digital contexts. Specifically, self-traits and device traits will influence customer's emotions, values and behaviors through the mediating mechanism of the immersive MAR app-enhanced experience.

### 6.2 Theoretical implications

Despite the importance of the immersive experience and the increasing research attention devoted to the phenomenon as summarized in Table 1, extant research is lacking in some aspects. First of all, the relative impacts of mobile apps, especially MAR apps in marketing domains, are still limited. In addition, to our knowledge, studies related to immersive MAR app-enhanced experiences are even more limited. Thus, it is essential to study the immersive experience in addition to how MAR apps can provide more information about products. Secondly, prior studies have demonstrated that not only individual traits (e.g. mental imagery, personal innovativeness), but also device traits consisting of environmental embedding and simulated physical control have connections with customers' immersive experiences (Renner et al., 2019; Ahmad and Abdulkarim, 2019; Hilken et al., 2017). Advanced technologies (e.g. MAR apps) can be a crucial tool for firms in enhancing the immersive customer experience (Hilken et al., 2020). Therefore, this study proposes a conceptual model showing the mediating role of the immersive experience on the linkages from customer self-traits and device traits to customer emotions, values and behavioral responses. *Thirdly,* recent research has emphasized that virtual technologies (i.e., MAR apps) can enhance customer emotions and decision-making evaluations (Dacko, 2017; Hilken et al., 2017). However, studies related to the immersive experience in technological advancements, such as MAR apps, as well as its subsequent impact on customer responses are still neglected (Javornik, 2016). Comprehension of the immersive experience can help firms gain higher performance by influencing customers' positive emotions (e.g., pleasure, arousal, dominance), customer values (e.g., utilitarian value, hedonic value, social value) and eventually changing their behavioral responses (e.g., purchase intention, word-of-mouth) (Novak et al., 2000; Yim et al., 2017). A broader understanding of customer responses after experiencing virtual environments is needed for marketing practitioners to develop more effective strategies for satisfying customers.

### 6.3 Practical implications

MAR apps are considered to be one of the most interactive and rapidly emerging technologies, and as such this study gives some suggestions related to MAR apps for practical makers. Firstly, digitalization (e.g. MAR apps) not only leads to changes in business activities (Parvinen et al., 2015), but also achieves competitive capacities (Brynjolfsson et al., 2013). Based on the potential of MAR apps, firms can provide more information to satisfy their customers. The immersive experience, the “experience of total engagement where other attentions, in essence, are ignored”, has recently gained attention from firms to attract their customers because of its role in influencing customer attitudes and behavioral responses (Agarwal and Karahanna, 2000). Therefore, there is a call for studying the immersive MAR app-enhanced experience, in particular the driving forces of the immersive experience and how it can influence customer responses. *Secondly,* this study emphasizes that virtual technologies (i.e., MAR apps) can provide more information and stimulate the immersive customer immersive (e.g., Yim et al., 2017), and thus they are also expected to affect customers' emotions and decision-making evaluations (Dacko, 2017; Hilken et al., 2017). However, the immersive experience in technological advancements, such as MAR apps and their impact on customer responses, are still neglected. The enhancement of the immersive experience can help firms achieve better performance by influencing customers' positive emotions (e.g., pleasure, arousal, dominance), customer values (e.g., utilitarian value, hedonic value, social value) and eventually changing their behavioral responses (e.g., purchase intention, word-of-mouth) (Novak et al., 2000; Yim et al., 2017). A broader understanding of customer responses after experiencing virtual environments is needed for marketing practitioners to develop more effective strategies to satisfy their customers.

## 7 Conclusions

Technological advancement has considerably transformed the business landscape; enhancing the immersive customer experience is an important means for firms to attain higher performance through influencing consumers' positive attitudinal and behavioral responses. Therefore, by systematically reviewing the existing literature on the antecedents, consequences, and contingencies of immersive experiences, the authors propose an integrated conceptual model (Figure 3) that describes the self-traits and device traits – immersive experience – responses linkages across MAR apps. The integrated framework offers managers and researchers a comprehensive understanding of the impacts of self-traits and device traits on the immersive experience, which in turn leads to customer responses in the virtual environments of MAR apps. Future research can empirically test a part or the entirety of the conceptual framework.

## Declarations

### Author contribution statement

Vo Kim Nhan: Analyzed and interpreted the data; wrote the paper.

Assoc. Prof. Dr. Le Thanh Tam: analyzed and interpreted the data; analysis tools or data; wrote the paper.

Assoc. Prof. Ho Tien Dung: analyzed and interpreted the data; wrote the paper.

Nguyen Thanh Vu: analyzed and interpreted the data; wrote the paper.

Assoc. Prof. Dr. Angelina Nhat Hanh Le: Shared the valuable knowledge and useful comments.

Huong Xuan Ho: Wrote the introduction part of paper, gave feedback and new ideas.

### Funding statement

This research is funded by the Ministerial-level Research Project No. B2021.KHA.04: “The study of conditions for developing fintech ecosystem to promote financial inclusion in Vietnam”.

### Data availability statement

No data was used for the research described in the article.

### Declaration of interests statement

The authors declare no conflict of interest.

### Additional information

No additional information is available for this paper.
